# Attitudes of emergency healthcare workers on electric vehicle accidents: a descriptive study

**DOI:** 10.1186/s12873-025-01408-4

**Published:** 2025-12-23

**Authors:** Uğur Kayhan, Zafer Liman, Şerife Özdinç, İbrahim Kılıç, Cengiz Durmuş

**Affiliations:** 1 Faculty of Medicine, Department of Forensic Medicine, Afyonkarahisar Health Sciences University, Afyonkarahisar, 03030 Turkey; 2 Faculty of Medicine, Department of Forensic Medicine, Karabuk University, Karabuk, 78050 Turkey; 3 Faculty of Medicine, Department of Emergency Medicine, Afyonkarahisar Health Sciences University, Afyonkarahisar, 03030 Turkey; 4 Faculty of Veterinary Medicine, Department of Biostatistics, Afyon Kocatepe University, Afyonkarahisar, 03200 Turkey; 5 Ambulance Service, Konya Provincial Directorate of Health, Konya, 42130 Turkey

**Keywords:** Electric vehicles, Electric vehicle accidents, Healthcare workers, Attitude scale, Prehospital emergency care

## Abstract

**Purpose:**

This study aimed to determine the cognitive, affective, and behavioral attitudes of healthcare professionals involved in the initial intervention, treatment, and reporting of electric vehicle accidents (EVAs) and to compare these attitudes on the basis of their individual characteristics.

**Design and methods:**

A descriptive cross-sectional design was employed. The sample consisted of 402 healthcare workers in Turkey. Data were collected via a survey that included demographic questions and the Electric Vehicle Accident Attitude Scale, a 27-item instrument developed by Kayhan et al. Attitudes were measured across cognitive, affective, and behavioral dimensions. Descriptive statistics (frequency, percentage, mean, and standard deviation) were used alongside t tests and ANOVA, and MANOVA to compare attitudes by personal and professional variables. Cluster analysis was used to group participants according to their EVA-related attitudes.

**Results:**

The participants demonstrated moderate overall attitudes toward EVAs. While cognitive and affective attitudes were low, behavioral attitudes were relatively high. No significant differences were found profession, marital status, age, or years of experience (*p* > 0.05); however, attitudes varied significantly by work unit and gender (*p* < 0.05). Compared with dispatch and emergency department staff, ambulance teams had more favorable behavioral (*M* = 3.77) and overall (*M* = 3.15) attitudes.

**Conclusions:**

EVAs pose unique risks due to their battery structure and limited control, distinguishing them from conventional vehicle incidents. Knowledge gaps and emotional hesitation among healthcare workers highlight the need for targeted training programs that extend beyond healthcare providers to include other frontline responders, such as firefighters and traffic police. In addition, forensic specialists involved in injury assessment and cause-of-death determination should receive EVA-specific awareness training to enhance their preparedness for such cases. Furthermore, the implementation of structured EVA-specific training programs—shaped by the attitudes identified in this study—may not only improve the quality of emergency care but also serve a preventive function in potential future legal risks related to patient care.

## Introduction

Traffic accidents remain one of the leading causes of death among children and young adults and impose a substantial economic burden on countries [1]. By 2030, traffic accidents are projected to become the fifth leading cause of death worldwide, underscoring the urgent need for preventive measures. Within this context, the technological transformation in transportation, particularly the increasing use of electric vehicles (EVs), has introduced new risk factors for traffic safety [2, 3]. In 2024, electric and hybrid vehicles accounted for approximately 12% of total vehicle sales in Turkey, whereas their global share exceeded 30%, with projections indicating a potential increase to 25–30% by 2025 [4, 5]. Recent reports suggest that EV-related crashes show distinctive patterns compared with conventional vehicles, as EVs carry a nearly 50% higher risk of vehicle damage although occupants may face a lower risk of injury, while new EV drivers are reported to be up to three times more likely to be involved in collisions due to unfamiliar acceleration, and EVs have also been linked to a higher risk of pedestrian accidents [6]. Although EV-related crashes still constitute a relatively small proportion of total accidents (e.g., ~ 7% in a 2023 regional dataset), they account for a disproportionate share of fatalities (~ 12% of total deaths in that sample) [7]. These trends underscore the growing importance of EV-specific risks in traffic accidents and the necessity of developing tailored emergency response protocols and preventive strategies. In light of these risks, enhancing the preparedness of emergency medical services has become imperative, and the early and effective intervention of teams is of vital importance.

The growing prevalence of electric vehicles in traffic also introduces unique injury mechanisms that differ from those observed in conventional car crashes. In EV-related crashes, the primary causes of injury are largely associated with the vehicle’s sudden acceleration dynamics and the damage sustained by batteries following impact or collision. In high‑energy crashes, these factors have been linked to a markedly increased risk of severe and complex trauma [8–12]. These risks include not only high-voltage electrical injuries and thermal burns from battery fires that are notoriously difficult to extinguish, but also blast-related injuries from battery explosions, inhalation of toxic fumes, and secondary chemical burns caused by electrolyte leakage [12–14]. Furthermore, delayed ignition or re‑ignition of damaged batteries has been reported, posing ongoing hazards even after the initial collision. Such risks significantly complicate on‑scene emergency response and require specialized training, protective equipment, and standardized protocols to ensure the safety of both patients and emergency personnel.

From a different perspective, forensic medicine plays a critical role in the comprehensive evaluation of traffic accidents [15]. Its primary functions include the medicolegal assessment of injury patterns, determination of the cause and manner of death, and preparation of forensic reports [16]. Furthermore, it plays an active role in both legal and medical proceedings by undertaking responsibilities such as conducting malpractice evaluations in lawsuits arising from emergency medical interventions related to traffic accidents.

Despite the growing number of EVs in traffic and their unique injury mechanisms, little is known about how prepared emergency healthcare workers feel to manage EVA-related risks. Attitudes—including knowledge, perceptions of risk, and behavioral tendencies—are critical, as they directly influence decision-making and the effectiveness of first response. Understanding these attitudes can therefore provide valuable insights into training needs and preparedness gaps. Therefore, this study aims to assess the knowledge, attitudes, and behaviors of emergency medical teams and emergency department staff regarding electric vehicle accidents (EVAs) and to utilize the findings to inform and enhance training programs.

## Methods

### Study design

This study has a descriptive design, as it seeks to present the current attitudes of emergency healthcare workers, and was conducted within a cross-sectional framework.

### Participants

The population of this study consisted of healthcare workers [Emergency medical technician (EMT), paramedics, physicians, and paramedics or EMT who also serve as ambulance drivers] from the Emergency Medical Services call center (ECC), ambulances, and hospital emergency departments in Turkey, who intervene in all vehicle accidents, including EV accidents, and are over 18 years of age. EMTs are at least graduates of health vocational high schools, paramedics have completed associate degree–level health education, and the ambulance drivers included in the study possess a basic first aid certificate and are graduates of either health vocational high schools or associate degree health programs. In addition, general practitioners, emergency medicine specialists, and nurses play an active role in the delivery of emergency healthcare, particularly at the hospital stage.

According to the most recently published official data in Turkey, it has been reported that a total of 33,900 personnel are employed in emergency medical services [17]. Due to constraints such as time, cost, a nationwide sample was recruited digitally through professional associations, occupational organizations, and social media groups with active membership across the country. Inclusion criteria were being 18 years or older and currently working as an active emergency healthcare professional who may intervene in traffic accidents, including EVAs; exclusion criteria were incomplete, contradictory, or unreliable survey responses, and individuals not actively employed in emergency healthcare services.

The minimum sample size was calculated using the formula n = s²Zα²/d². Based on a pilot study with 30 participants (standard deviation = 1), a significance level of 0.05 (Z₀.₀₅ = 1.96), and an effect size of 0.1, the required sample size was estimated as 384 [18]. A total of 410 responses were collected. Following data quality checks, 8 questionnaires were excluded due to inconsistent or unreliable patterns (e.g., identical responses to all items). Although the online platform prevented incomplete submissions, exclusions were necessary in cases with contradictory or illogical answers. The final analysis was therefore conducted on 402 participants. The prepared questionnaire was hosted to an online platform, and the access link was distributed. Response rate could not be calculated due to online recruitment; however, participants represented a broad geographic and professional distribution via nationwide professional associations and occupational organizations, which suggests coverage beyond a single region, although the exact geographic distribution could not be determined. The online platform was designed to require responses to all items, minimizing missing data. The survey took approximately 15 min to complete, and no identifying information (e.g., names or email addresses) was collected.

### EVAAS measures and scoring

The data collection technique used in the study was a two-part questionnaire. The first section included 8 closed-ended questions about the participants’ individual characteristics (work unit, profession, gender, marital status, age, work experience, training on EV accidents, and intervention in such accidents). In the second section, the Electric Vehicle Accident Attitude Scale (EVAAS), a 27-item instrument developed and validated by Kayhan et al. [19]. The scale is structured into three dimensions: cognitive (12 items), affective (8 items), and behavioral (7 items). Each item is rated on a 5-point Likert scale ranging from “Strongly Disagree = 1” to “Strongly Agree = 5,” with negatively worded items reverse-coded (Strongly Disagree = 5 to Strongly Agree = 1). Subscale and overall scores were calculated as mean values, assuming equal weight for each item, with higher scores reflecting more positive attitudes. Internal consistency was satisfactory, with Cronbach’s Alpha coefficients above 0.80 for all subscales and 0.862 for the overall scale. Validity and reliability were established through Delphi technique, focus group evaluation, and pilot testing, and the final version explained approximately 65% of the total variance. Sample items include: “*I have sufficient knowledge about EVAs and risk factor”* (cognitive), “*I feel psychologically prepared for EVAs*” (affective), and “*I use protective equipment (gloves*,* shoes*,* etc.) when responding to EVAs due to electric shock and thermal injuries*” (behavioral), and the full scale items are presented in Table 2.

### Statistical analysis

The data obtained in the study were analyzed via SPSS 21.0 for Windows (SPSS, Inc., Chicago: SPSS Inc. PASW Statistical Program. Version 21.0 Chicago, IL, USA: SPSS Inc.; 2009). Descriptive statistics, such as frequency, percentage, arithmetic mean, and standard deviation, were used in the data analysis. The normality of the participants’ cognitive, affective, behavioral, and overall attitude scale scores was examined via the Kolmogorov‒Smirnov test, which revealed a normal distribution (Z = 1.023; *p* > 0.05). In this context, a Multivariate Analysis of Variance (MANOVA) was conducted to compare cognitive, affective, behavioral, and overall attitude scores across demographic characteristics. Tukey’s test was used for pairwise group comparisons, and effect sizes were determined using eta squared values. Given the unequal sample sizes between work units (ambulance crew = 364, ECC = 15, emergency department = 23), Welch’s ANOVA with Games–Howell post hoc tests was applied to ensure robustness against heterogeneity of variances. For all comparisons, 95% confidence intervals and effect sizes (eta squared) were reported.

Cluster analysis was conducted to identify and classify individual-level attitudes of emergency healthcare workers toward EVs and EVAs, as well as to determine the groups and individuals who reported low levels of attitudes in the cognitive, affective, and behavioral dimensions and who may therefore require additional support. The analysis was performed using Ward’s method with Euclidean distance based on participants’ overall attitude scale scores. Subsequently, an Analysis of Variance (ANOVA) was conducted to compare the attitude scores across the obtained clusters. The statistical significance level for all tests was set at *p* < 0.05.

## Results

The demographic characteristics of the 402 healthcare workers who participated in the study are presented in Table 1. A total of 90.5% of the participants worked as Ambulance crew, 85.6% were EMTs and paramedics, 56.8% were male, 71.1% were unmarried, 87.5% were aged between 25 and 45 years, 39.3% had 11–15 years of work experience, and 94.5% had not received training on electric vehicle accidents.

**Table 1:** Distribution of participants according to demographic characteristics (*n* = 402)

Variables	Groups	*n*	%
Unit	Ambulance crew	364	90.5
	ECC*	15	3.7
	Emergency department staff	23	5.8
Profession	EMT**	170	42.3
	Paramedic	174	43.3
	Medical Doctor	18	4.4
	Ambulance driver	40	10.0
Gender	Female	176	43.8
	Male	226	56.8
Marital status	Married	116	28.9
	Single	286	71.1
Age (year)	< 25	30	7.5
	25–35	180	44.8
	36–45	172	42.7
	45 >	20	5.0
Years of professional experience	< 10	84	20.9
	11–15	158	39.3
	16–20	89	22.1
	20 >	71	17.7

*ECC: Emergency Medical Service Call Center. ** EMT: Emergency Medical Technician

Descriptive statistics regarding healthcare workers’ attitudes toward electric vehicle accidents are presented in Table 2. The overall attitudes of the healthcare workers in the sample group are moderate on the 5-point Likert scale (*M* = 3.13). The results revealed that the cognitive (*M* = 2.90) and affective (*M* = 2.95) attitudes of the participants toward electric vehicle accidents were lower than their behavioral attitudes (*M* = 3.74). An examination of the item means for each dimension in Table 2 revealed that healthcare workers’ knowledge was particularly low in the cognitive dimension regarding the electrical components of EVs (*M* = 1.66), their working systems (*M* = 1.77), risk factors related to electric vehicle accidents (*M* = 1.79), preventive measures (*M* = 1.88), and the identification of the vehicle’s electric status during an accident (*M* = 2.19). In the affective dimension, the levels of fear of EV accidents, hesitation in the intervention, discomfort due to lack of knowledge, and concern about battery-related injuries were moderate. In the behavioral dimension, the attitudes of the participants were found to be above average for all items.

**Table 2:** Descriptive statistics regarding healthcare workers’ attitudes toward evas

Items	M ± SD
***Cognitive Dimension (1–12)***	***2.90 ± 0.57***
1. The types and risks of traffic accidents vary depending on the vehicle fuel types.	4.15 ± 1.15
2. I do not believe that EVAs are different from other traffic accidents.	3.62 ± 1.36
3. I have sufficient knowledge about the operational system of EVs (battery. current. etc.).	1.77 ± 0.99
4. I can identify the vehicle involved in the accident as an EV based on characteristics such as brand. model. logo. etc.	2.19 ± 1.30
5. I have sufficient knowledge about the electrical components of EVs (battery. cables. etc.).	1.66 ± 0.97
6. I have sufficient knowledge about EVAs and their risk factors.	1.79 ± 1.04
7. I am aware of the measures that can be taken against EVAs.	1.88 ± 1.07
8. Fires caused by batteries in EVAs are very difficult to extinguish.	3.22 ± 1.33
9. I am aware that battery-related toxic gases (methane. cyanide. etc.) in EVAs can cause poisoning.	2.92 ± 1.29
10. Battery-related explosive injuries may occur in EVAs.	3.68 ± 1.14
11. Battery-related electrical shocks or thermal injuries may occur in EVAs.	3.84 ± 1.08
12. Standard equipment should be developed for intervention in EVAs	4.18 ± 1.06
***Affective Dimension (13–20)***	***2.95 ± 0.81***
13. I feel psychologically prepared for intervention in EVAs.	3.46 **±** 1.41
14. The knowledge I have about EVAs reassures me.	3.59 **±** 1.38
15. I possess the necessary attention and concentration in the face of the high risks in EVAs.	3.39 **±** 1.39
16. EVAs generally scare me.	2.88 **±** 1.34
17. I hesitate to intervene in EVAs.	3.01 **±** 1.38
18. My lack of knowledge about EVAs makes me uneasy.	2.61 **±** 1.34
19. Fires that are difficult to extinguish in EVAs scare me	2.43 **±** 1.25
20. I am concerned about battery-related explosions. toxic gases. and thermal injuries in EVAs.	2.26 **±** 1.19
***Behavioral Dimension (21–27)***	***3.74 ± 0.78***
21. I pay attention to the type of fuel of the vehicle when intervening with injured individuals in EVAs.	3.53 ± 1.24
22. I attend training sessions on EVAs and related injuries.	3.72 ± 1.46
23. I do not intervene in EVA accidents until the rescue team-firefighters arrive.	3.68 ± 1.33
24. I use protective equipment against electrical shocks and thermal injuries when intervening in EVAs.	4.09 ± 1.16
25. I use a gas mask due to the suspicion of toxic gas release from the batteries when intervening in EVAs.	3.07 ± 1.48
26. I take precautions for myself and the injured individuals when intervening in EVAs.	4.26 ± 0.99
27. Due to the silent operation of EVs and their difficulty in being noticed. I approach the vehicle from the side to avoid the possibility of sudden movement in accidents.	3.85 ± 1.10
**General Attitude (1–27)**	**3.13 ± 0.46**

EVA: electric vehicle accident. EV: electric vehicle. M: mean; SD: standard deviation

To reveal the multivariate effects of demographic variables on cognitive, affective, behavioral, and overall attitude scores, a MANOVA was conducted, and the statistically significant findings were presented in Table 3. The attitudes of the participants toward these accidents did not significantly differ according to profession, marital status, age, or years of work experience (*p* > 0.05), whereas statistically significant differences were found according to the unit worked and gender (*p* < 0.05), as shown in Table 3. The unit*gender interaction was not found to be statistically significant as a result of the MANOVA. Accordingly, the behavioral attitudes (*M* = 3.77) and general attitudes (*M* = 3.15) of the ambulance team, which performs the first medical intervention in the field, were found to be more positive than those of the ECC and emergency department workers. However, while no difference was found in the behavioral dimension, males had greater cognitive (*M* = 3.01), affective (*M* = 3.06), and general attitudes (*M* = 3.21) than females did.

**Table 3:** Comparison of healthcare workers’ attitudes toward evas based on demographic characteristics

Variables	Groups	Cognitive	Affective	Behavioral	General Attitude
		M ± SD (CI)	M ± SD (CI)	M ± SD (CI)	M ± SD (CI)
Unit	Ambulance crew	2.92 ± 0.56 (2.86–2.98)	2.96 ± 0.81 (2.87–3.04)	3.77 ± 0.78 ^a^ (3.69–3.85)	3.15 ± 0.45 ^a^ (3.10–3.20)
	ECC**	2.83 ± 0.78 (2.40–3.27)	2.79 ± 1.01 (2.23–3.35)	3.45 ± 0.60 ^b^ (3.12–3.79)	2.93 ± 0.49 ^b^ (2.65–3.20)
	Emergency department staff	2.72 ± 0.58 (2.46–2.97)	2.89 ± 0.66 (2.60–3.18)	3.40 ± 0.74 ^b^ (3.08–3.73)	2.95 ± 0.43 ^b^ (2.76–3.13)
	P	0.204	0.681	0.033*	0.024*
	Eta Square	0.008	0.002	0.017	0.019
Gender	Female	2.78 ± 0.52 (2.69–2.84)	2.80 ± 0.78 (2.69–2.92)	3.73 ± 0.73 (3.62–3.84)	3.03 ± 0.44 (2.96–3.09)
	Male	3.01 ± 0.59 (2.93–3.09)	3.06 ± 0.81 (2.95–3.17)	3.74 ± 0.82 (3.63–3.85)	3.21 ± 0.44 (3.15–3.27)
	P	0.023*	0.022*	0.923	0.024*
	Eta Square	0.017	0.013	0.001	0.018
Unit*Gender	P	0.168	0.225	0.838	0.258
	Eta Square	0.009	0.008	0.001	0.007

^*^P < 0.05; ^a.b^ There are significant differences between groups with different letters (*p* < 0.05). **ECC: Emergency Medical Service Call Center; CI: Confidence Interval

In this study, cluster analysis was performed to classify healthcare workers’ attitudes toward EVA, resulting in a total of four clusters (Table 4). The first cluster consisted of 21.6% of the participants, the second cluster included 27.9%, the third cluster contained 28.1%, and the fourth cluster made up 22.4%. The first cluster, which was based on cognitive, affective, and behavioral dimensions and general attitudes, represented the most positive group and was therefore labeled “GOOD.” Accordingly, the second group was named “NO BAD,” the third group was labeled “ATTENTION,” and the fourth group, which exhibited the most negative attitudes, was named the “ALARM” group.

**Table 4:** Comparison of the attitudes of the four groups formed as a result of cluster analysis

Clusters		Cognitive	Affective	Behavioral	General Attitude
	*n* (%)	M ± SD (CI)	M ± SD (CI)	M ± SD (CI)	M ± SD (CI)
1. Good	87 (21.6)	3.30 ± 0.29 ^a^ (3.22–3.39)	3.72 ± 0.55 ^a^ (3.60–3.83)	4.28 ± 0.48 ^a^ (4.19–4.35)	3.47 ± 0.23 ^a^ (3.42–3.52)
2. No bad	112 (27.9)	3.00 ± 0.56 ^b^ (2.93–3.13)	3.19 ± 0.54 ^b^ (3.07–3.31)	4.00 ± 0.42 ^b^ (3.90–4.11)	3.27 ± 0.35 ^b^ (3.20–3.34)
3. Attention	113 (28.1)	2.83 ± 0.52 ^c^ (2.76–2.91)	2.67 ± 0.63 ^c^ (2.56–2.77)	3.49 ± 0.64 ^c^ (3.29–3.69)	3.12 ± 0.38 ^c^ (3.05–3.19)
4. Alarm	90 (22.4)	2.40 ± 0.56 ^d^ (2.28–2.52)	2.25 ± 0.74 ^d^ (2.09–2.40)	3.19 ± 0.95 ^d^ (3.07–3.31)	2.64 ± 0.39 ^d^ (2.55–2.72)
P		0.001^*^	0.001^*^	0.001^*^	0.001^*^
Eta Square		0.001	0.001	0.001	0.001

^*^P < 0.01 ^a. b. c. d^ There are significant differences between groups that contain different letters (*p* < 0.05). CI: Confidence Interval

## Discussion

Although the initial trauma sustained in traffic accidents is the primary factor affecting mortality and morbidity, the first medical response process to the injury is also a significant determinant [20]. In the context of EVAs, the unique characteristics of these vehicles introduce additional challenges for first responders [8]. In our study conducted with emergency healthcare personnel using the EVAAS scale developed by Kayhan et al. [19] to evaluate the impacts of these challenges, the most notable finding was that healthcare workers demonstrated moderate overall attitudes toward EVAs. Cognitive and affective dimensions—reflecting knowledge, fear, and anxiety—scored lower than behavioral attitudes, suggesting that healthcare workers are neither completely unprepared nor ideally equipped to respond to EVAs.

In terms of work unit, 90.5% of emergency healthcare personnel were members of ambulance crews. Subgroup analyses indicated that ECC and hospital emergency department staff exhibited more negative overall and behavioral attitudes compared with ambulance personnel. This difference may be attributed to the unique role of ambulance teams, who are the first to arrive at the accident scene and are therefore more accustomed to crisis management and rapid decision-making in dynamic environments [21, 22]. MANOVA analyses revealed that the unit variable, in combination with gender, had a joint effect on cognitive, affective, and behavioral dimensions, with female ECC staff scoring lowest across subgroups.

In terms of profession, 85.6% were EMTs or paramedics, and 28.9% were married. No significant differences in attitudes were found based on profession or marital status. This finding may reflect the relative novelty of EVAs and the presence of similar knowledge gaps across all professional roles.

Female participants demonstrated more negative cognitive, affective, and overall attitudes than male participants. This difference may be partially explained by sociocultural and experiential factors, such as men’s greater likelihood of early and frequent exposure to vehicles, which can increase familiarity and confidence, whereas women may approach such scenarios with greater caution [23–25]. Literature also indicates that women tend to exhibit stronger emotional responses to stress and emergencies and report higher levels of perceived risk across various domains, including health, environmental hazards, and technology-related risks [23, 24].

No significant association was found between age and attitude scores. While previous research has reported positive effects of age on emergency management skills, this relationship may not have emerged in the present study due to the novelty of EVAs, which limits the influence of age-related experience advantages [26].

Years of professional experience did not significantly affect attitudes toward EVAs. The limited occurrence of EVAs may restrict even highly experienced personnel from developing the specific knowledge and practical familiarity necessary for confident management of such incidents.

When the findings of the study are considered as a whole, they suggest a need for improvement across all professional groups, particularly in cognitive and affective dimensions—knowledge, fear, and anxiety. Standardized EVA-specific training programs covering all roles would be beneficial. These should include foundational technical knowledge of EV mechanics, battery-related hazards, and safe intervention methods, combined with simulation-based training and scenario-driven exercises to enhance practical skills. Training should also address emotional barriers such as fear and hesitation [21, 25]. High-risk subgroups, especially ECC personnel and female healthcare workers, may warrant being prioritized. Participants classified in the ATTENTION and ALARM clusters in the exploratory analysis could benefit from more intensive, hands-on, and confidence-building training.

In addition to recognizing that the most important step is the implementation of comprehensive training, policies that strengthen interagency coordination and ensure standardized field procedures are also required. These policies should primarily address frontline healthcare providers involved in acute EVA management (including emergency department physicians, physicians from other specialties, and allied healthcare personnel involved in hospital processes), but they must also cover professionals with secondary responsibilities such as forensic medicine specialists. Forensic medicine specialists contribute significantly to the legal process by identifying various EVA-related injury mechanisms and incorporating these findings into accurate and comprehensive medicolegal reports. In fatal cases, their expertise is critical for determining the exact cause and manner of death, and in malpractice assessments, for evaluating whether delays or errors in emergency interventions occurred and whether these affected patient outcomes. Comprehensive strategies should therefore include the development of EVA-specific response protocols, certification programs, and the integration of EVA-focused modules into emergency healthcare curricula, while also extending to non-healthcare first responders such as traffic police, firefighting units, search and rescue teams, and vehicle maintenance personnel. Collectively, these strategies may enhance both the safety of responders and the quality of patient outcomes in the context of EVAs. International frameworks, such as those developed by the National Fire Protection Association [27] and the Occupational Safety and Health Administration [28], provide structured guidance on responder safety in incidents involving high-voltage systems. Adapting and integrating such standards into national EVA training curricula could further strengthen preparedness and interagency coordination.

## Conclusion

In conclusion, the findings of this study highlight the importance of providing EVA-specific training for emergency healthcare workers. This need can be extended to other relevant professional groups involved in EVA management. To address this, effective cooperation between healthcare institutions, law enforcement agencies, fire departments, and regulatory authorities is essential to ensure coordinated and context-appropriate interventions.

## Limitations

This study has certain limitations. The use of self-reported questionnaires may have introduced recall or social desirability bias. Although participants were recruited from across Turkey, the response rate could not be calculated and representativeness is therefore not guaranteed. Moreover, because EV technology is still novel and real-life EVA encounters remain relatively rare, participants’ attitudes may have been shaped by limited direct experience rather than professional competencies. In addition, the reliance on a questionnaire-based survey restricts the ability to capture actual performance in real emergency scenarios. Future research should evaluate the effectiveness of these strategies, including simulation-based training and innovative tools such as virtual reality, to strengthen knowledge and improve both occupational safety and patient outcomes in the context of EVAs.

## Data Availability

The datasets used and analyzed during the current study are available from the corresponding author upon reasonable request.

## Abbreviations

EMT: Emergency medical technician
ECC: Emergency Medical Service Call Center
EVAAS: Electric Vehicle Accident Attitude Scale
EV: Electric Vehicle
EVA: Electric Vehicle Accident
M: Mean
SD: Standard deviation
et al.: Et alia (and others)
